# Rare presentation of primary varicella zoster as fatal fulminant hepatitis in adult on low-dose,short-term steroid: Case report

**DOI:** 10.1016/j.amsu.2019.10.034

**Published:** 2019-11-09

**Authors:** Ali Toffaha, Walid El Ansari, AF. Ramzee, Mohammad Afana, Hesham Aljohary

**Affiliations:** a Department of General Surgery, Hamad Medical Corporation, Doha, Qatar; b Department of Surgery, Hamad General Hospital, Hamad Medical Corporation, Doha, Qatar; c College of Medicine, Qatar University, Doha, Qatar; d School of Health and Education, University of Skövde, Skövde, Sweden; e Department of Internal Medicine, Hamad Medical Corporation, Doha, Qatar

**Keywords:** Fulminant acute hepatic failure, Biliary colic, Case report, Liver failure, Hepatitis

## Abstract

**Background:**

Varicella zoster virus presents clinically as primary (chickenpox) or secondary (herpes zoster) infection. Cutaneous and extracutaneous dissemination may occur, usually in immunocompromised patients. VZV hepatitis that progresses to fulminant hepatic failure is very rare and fatal. To the best of our knowledge, 9 cases have been reported to date, of which 7 were in immunocompromised adults, and only one patient was on short duration steroid therapy.

**Presentation of case:**

We present a 26-year old man who was admitted initially with acute abdomen as query persistent biliary colic. Later, he showed clinical and laboratory findings of VZV hepatitis that progressed rapidly despite maximal medical ICU support and he expired on day 3 of admission.

**Conclusions:**

Acute VZV infection may present as fulminant hepatitis. The presentation may initially be challenging for the diagnosis and should be considered if the patient has been in contact with a sick case. Low dose corticosteroid could carry a risk for fatal VZV fulminant hepatitis and should be used very cautiously especially with VZV patients’ contacts. Further causative relationships remain to be established.

## Highlights

• Acute VZV infection may present as fulminant hepatitis initially without rash.
• Presentation may initially be challenging for the diagnosis.
• Low dose corticosteroid could carry a risk for fatal VZV fulminant hepatitis.
• VZV hepatitis may be rapidly progressive and fatal.

## 1 Background

Varicella zoster virus presents clinically as primary (chickenpox) or secondary (herpes zoster) infection. Primary infection typically produces generalized vesicular rash. The virus then establishes latency in the dorsal root ganglia and may later reactivate as secondary infection, generating a localized dermatomal “shingles” vesicular eruption. Cutaneous and extracutaneous dissemination can occur, mostly in immunocompromised individuals [1]. Fulminant acute hepatic failure (FAHF) due to VZV hepatitis is extremely rare and deadly, with only 9 cases reported in the literature. Seven of these nine cases were immunocompromised patients, one was 15 year old child and only one case was on short term steroid treatment; most of them died of the disease [[2], [3], [4], [5], [6], [7], [8], [9], [10]]. In this paper, we present, to the best of our knowledge, the second case of VZV FAHF to develop in a healthy adult on low dose steroid that unusually presented initially as acute abdomen. We report this case in line with the updated consensus-based surgical case report (SCARE) criteria [11].

## 2 Case presentation

A 26-year old man presented to the emergency department at Hamad Medical Corporation (HMC) in Doha, Qatar, with a three-day history of severe colicy epigastric and right upper quadrant abdominal pain, radiating to the back, associated with two episodes of vomiting and constipation. This was his first time to have such pain. He had past surgical history of open appendectomy during adolescence and was currently on oral medications for sciatica (prednisolone 5 mg BID for one-week, Naproxen 225 mg BID and Gabapentin 300 mg daily). Otherwise, he had no comorbidities, and the other systemic review was unremarkable. No recent travel or interaction with sick contacts was mentioned at the initial encounter at the emergency department.

The patient had normal vital signs. Abdominal examination revealed severe epigastric and right upper abdomen tenderness. Otherwise his examination was unremarkable at that time. Initial labs including CBC, complete metabolic panel were within normal range. Imaging including abdomen and chest x-rays, and CT angiogram abdomen were normal; however, US abdomen showed gallbladder sludge but with no signs of cholecystitis. Hence the patient was admitted by the surgical team with an initial diagnosis of possible persistent biliary colic.

During the night of admission day (day 0), the patient was still having severe colicy pain that required high doses of opioids, paracetamol and NSAIDs. Repeat set of all labs were undertaken and due to the colicy nature and persistent severe pain, a CT with oral contrast was done to exclude proximal bowel obstruction that might not have shown on the previous CT with IV contrast. The CT was normal, and the labs showed slightly rising transaminases (AST 94 IU, ALT 95 IU). The patient was kept nil per oral, on pain medications with IV hydration and a repeat set of labs were ordered to be done in 8 hours (early morning of day 1). During that time, the patient started to develop, for the first time, a few vesicles on the chest wall, his liver enzymes were rising (AST 375 IU, ALT 263 IU), and other labs were normal. When questioned specifically about having contact with someone with similar vesicular rash, the patient reported that one of his roommates had such eruption one week ago and did not seek medical advice as it improved on its own. The patient also did not remember having chickenpox during his childhood.

**Fig. 1:**
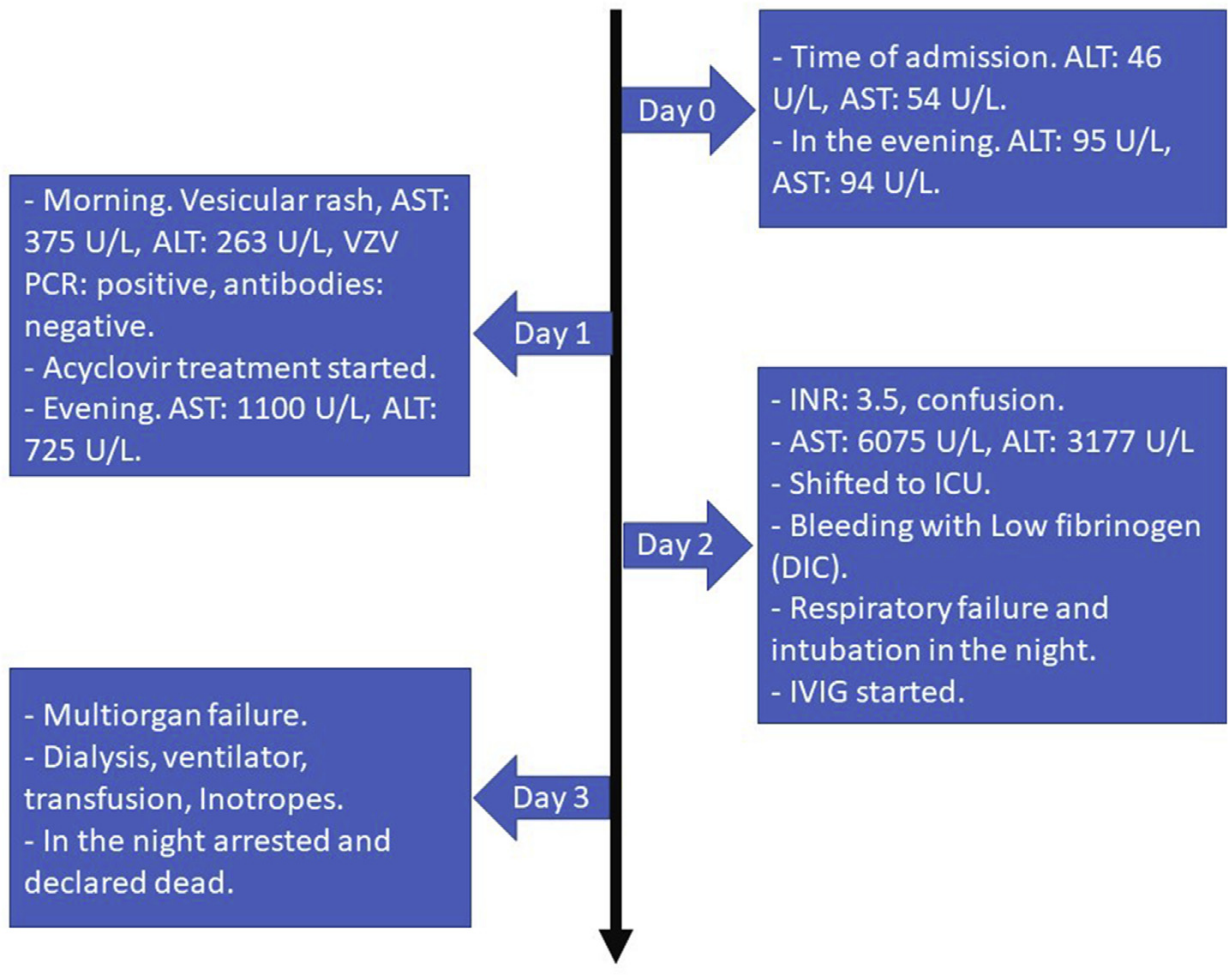
Sequence of events across 4 days.

The diagnosis of VZV hepatitis was immediately considered and the medical team was involved. Full hepatitis work up was undertaken on this admission day 1 during which the patient was still having severe pain, the rash progressed, and liver enzymes were rapidly rising. Viral panel came out to be positive for VZV PCR, but other (metabolic, toxicology, viral) work ups for hepatitis were negative. Thus, acyclovir was started by the infectious disease team. On day 2, the patient was shifted to the ICU as he was developing confusion, high INR, DIC and multiorgan failure that progressed rapidly despite all supportive measures. The patient died on day 3. Fig. 1 illustrates the sequence of events.

## 3 Discussion

VZV can affect both immunosuppressed and immunocompetent adults [6]. Dissemination of VZV infection and fulminant hepatitis happens in immunocompromised patients [[2], [3], [4], [5], [6], [7], [8], [9]]; but the infection may occasionally occur in immunocompetent patients due to extremely virulent strains that can be rapidly fatal [6]. The role of previous steroid treatment as a trigger for a temporary immunodepressed state must be considered [6]. Contrary to most cases reported in the literature, our patient was a healthy adult who was only on low dose oral steroid for his back pain. Steroids as a possible risk for disseminated VZV infection has been reported only in one case. In Italy, a patient on low dose steroid (prednisolone 5 mg BID for 3 days then 5 mg daily for another 3 days) for tonsillitis developed VZV FAHF [6]. The only difference between our case and the case in Italy was that their patient had positive HHSV6 in concomitance with his VZV infection, and it is not clear whether such coexistent infection could have added to the burden of the VZV infection driving it into a systemic disease involvement [6].

In terms of presentation, our patient initially presented with excruciating right upper quadrant pain and tenderness, without the typical vesicular rash of primary varicella infection which appeared later on day 1. This is in agreement with a case series (4 patients) where the initial severe abdominal pain appeared 2–4 days before the classical herpetic vesicular skin lesions (3 patients), while the fourth case never developed skin lesions [12]. Visceral VZV may present with abdominal pain with or without skin eruption [13], where the absence of skin lesions may delay timely diagnosis and treatment, particularly that abdominal imaging findings suggestive of the diagnosis of visceral VZV have rarely been reported [14].

In terms of diagnosis, we had a delay of one day due to the absence of typical rash and the initial normal liver function tests. Whilst such delay is consistent with other reports where the initial normal labs despite pain delayed identification of the disease [4], other cases had high liver enzymes at the first examination, but delays were still encountered due to the absence of rash [3]. Our patient started to show FAHF on Day 1. The differential diagnosis of FAHF includes venous obstruction, ischemia, toxins, medications, autoimmune hepatitis, metabolic and infectious causes (primarily viruses, e.g. hepatitis A-E, VZV, CMV, EBV, HSV, and adenovirus), with VZV being the rarest [10]. Our patient's history and clinical findings did not suggest ischemic hepatitis, and he did not have history of exposure to toxins or alcohol ingestion. A key clue was his contact history with a roommate with similar rash (probably chickenpox); had such information been evident earlier, it would have provided better direction towards the diagnosis.

Definitive diagnosis of VZV hepatitis can be made by liver biopsy, histopathology, culture, or VZV PCR [10]. Testing for VZV IgG might differentiate primary infection (negative IgG) from secondary reactivation (positive IgG) [4]. However, the lack of specificity and sensitivity of the VZV IgM antibodies render the test not very useful clinically in the differentiation of primary from recurrent VZV infection nor in the diagnosis [4]. PCR is the only readily available and sensitive (>99%) diagnostic tool, and should be used early in any suspicious case [4]. Our patient had negative VZV antibodies, but positive PCR confirmed the infection. Liver biopsy in the early stages is questionable, the disease may exhibit major or minor hepatic impairment but sometimes an etiologic diagnosis can be obtained by presence of nuclear inclusions [15]. Biopsy was not done in our case as PCR confirmed the diagnosis and post-mortem pathology was declined by the family.

In terms of treatment, the diagnosis of visceral VZV infection is often difficult due to atypical presentation, so treatment is often delayed [10]. Prompt empirical therapy with acyclovir and VZV immunoglobulin while awaiting PCR results is warranted if clinically suspected [16]. Other therapies include VZV immunoglobulin, liver transplant, IVIG, and supportive care [[2], [3], [4], [5], [6], [7], [8], [9]]. Since VZV AFHF has high mortality, VZV hepatitis should be considered in all patients with liver failure who present with rash and early treatment is critical. The timing of initiation of therapy is widely variable (range: first day of symptoms to >1 week) [10], and in some cases diagnosis was even post mortem and treatment was never initiated [10]. For our patient, the absence of initial diagnosis upon admission resulted in a one-day delay of acyclovir treatment; IVIG was started later on Day 2.

As for the outcomes, AFHF is very rare and deadly. Among 8 adult cases of VZV AFHF, only two survived [10]. Further studies on the rapid initial treatment of VZV AFHF and its outcome are required.

## 4 Conclusions

Acute VZV infection may present as AFHF, and the diagnosis may be challenging based on the initial presentation. Hence detailed contact history should be taken, and VZV should be considered when a patient has contact history with someone having VZV active infection. Low dose corticosteroid may carry a risk for VZV to progress to AFHF.

## Consent

Written informed consent was obtained from the patient's guardian (his brother as the patient had expired) for publication of this case report. A copy of the written consent is available for review by the Editor-in-Chief of this journal on request.

## Provenance and peer review

Not commissioned, Editor reviewed.

### Ethical approval

All the information was given retrospectively from the chart review and the patient was de-identified, this case report was approved by medical research center, Hamad Medical Corporation reference number (MRC-04-19-266).

## Funding

Nothing to declare.

## Author contribution

Ali Toffaha: study concept, data collection, interpretation, writing the paper, Walid El Ansari, data interpretation, writing the paper, A.F. Ramzee, data interpretation, writing the paper, Mohammad Afana, data interpretation, writing the paper, Hesham Aljohari, data interpretation, writing the paper.

## Registration of research studies

Name of the registry: researchregistry.

Unique Identifying number or registration ID: researchregistry5043.

Hyperlink to the registration (must be publicly accessible): https://www.researchregistry.com/register-now#home/registrationdetails/5d3c224113e2dd0010e38582/

## Guarantor

Ali Toffaha: Atoffaha2@gmail.com.

Walid El Ansari: welansari9@gmail.com.

## Declaration of competing interest

The authors declare no conflicts of interest.
